# Field-Effect Sensors for Virus Detection: From Ebola to SARS-CoV-2 and Plant Viral Enhancers

**DOI:** 10.3389/fpls.2020.598103

**Published:** 2020-11-24

**Authors:** Arshak Poghossian, Melanie Jablonski, Denise Molinnus, Christina Wege, Michael J. Schöning

**Affiliations:** ^1^MicroNanoBio, Düsseldorf, Germany; ^2^Institute of Nano- and Biotechnologies, FH Aachen University of Applied Sciences, Jülich, Germany; ^3^Institute of Pharmaceutical Chemistry, Philipps-University Marburg, Marburg, Germany; ^4^Institute of Biomaterials and Biomolecular Systems, University of Stuttgart, Stuttgart, Germany; ^5^Institute of Complex Systems (ICS-8), Research Centre Jülich GmbH, Jülich, Germany

**Keywords:** COVID-19, plant VLP tool, label-free detection, virus, field effect, biosensor, charged biomolecules, plant virus nanoparticle

## Abstract

Coronavirus disease 2019 (COVID-19) is a novel human infectious disease provoked by severe acute respiratory syndrome coronavirus 2 (SARS-CoV-2). Currently, no specific vaccines or drugs against COVID-19 are available. Therefore, early diagnosis and treatment are essential in order to slow the virus spread and to contain the disease outbreak. Hence, new diagnostic tests and devices for virus detection in clinical samples that are faster, more accurate and reliable, easier and cost-efficient than existing ones are needed. Due to the small sizes, fast response time, label-free operation without the need for expensive and time-consuming labeling steps, the possibility of real-time and multiplexed measurements, robustness and portability (point-of-care and on-site testing), biosensors based on semiconductor field-effect devices (FEDs) are one of the most attractive platforms for an electrical detection of charged biomolecules and bioparticles by their intrinsic charge. In this review, recent advances and key developments in the field of label-free detection of viruses (including plant viruses) with various types of FEDs are presented. In recent years, however, certain plant viruses have also attracted additional interest for biosensor layouts: Their repetitive protein subunits arranged at nanometric spacing can be employed for coupling functional molecules. If used as adapters on sensor chip surfaces, they allow an efficient immobilization of analyte-specific recognition and detector elements such as antibodies and enzymes at highest surface densities. The display on plant viral bionanoparticles may also lead to long-time stabilization of sensor molecules upon repeated uses and has the potential to increase sensor performance substantially, compared to conventional layouts. This has been demonstrated in different proof-of-concept biosensor devices. Therefore, richly available plant viral particles, non-pathogenic for animals or humans, might gain novel importance if applied in receptor layers of FEDs. These perspectives are explained and discussed with regard to future detection strategies for COVID-19 and related viral diseases.

## Introduction

Virus outbreaks remain one of the global problems of our time. Due to the increased mobility of populations as well as the sustained growth in international travel accelerated by globalization, a large number of viruses are spreading rapidly around the globe causing infectious disease outbreaks. Recent well-known examples are severe acute respiratory syndrome coronavirus (SARS-CoV, 2002–2003), H1N1 influenza A virus (swine flu, 2009–2010), Middle East respiratory syndrome coronavirus (MERS-CoV, 2012), Ebola virus (2014–2016), or Zika virus (2015–2016) (Choi et al., 2017; Lee et al., 2018). Moreover, viruses present a growing concern as potential agents for biological warfare and terrorism.

Coronavirus disease 2019 (COVID-19) is a novel human infectious disease provoked by severe acute respiratory syndrome coronavirus 2 (SARS-CoV-2) (Paraskevis et al., 2020; Seo et al., 2020; Weiss et al., 2020; Zhang and Holmes, 2020; Zhou et al., 2020). SARS-CoV-2 is highly contagious and has been widely spread worldwide, provoking public health crisis and an unprecedented socio-economic burden in most countries (Morales-Narváez and Dincer, 2020). Due to the rapid increase in the rate of human-to-human infection transmission, the World Health Organization (WHO) has classified the COVID-19 outbreak as a pandemic [as on March 11, 2020 World Health Organization [WHO], 2020 Situation Report-52]. As of November 02, 2020, the total number of confirmed COVID-19 cases around the world was more than 46 millions, resulting in more than 1.2 million deaths (WorldOmeter, 2020; COVID-19).

Currently, no United States Food and Drug Administration (FDA)-approved specific vaccines or curative drugs for the treatment of COVID-19 patients are available. Therefore, one of the key challenges in the effective fight against COVID-19 is the rapid and accurate identification of virus-infected patients (including asymptomatic patients), in order to apply appropriate protective measures (e.g., quarantine, isolation of patients in an early stage, and lockdown) and to slow the rate of transmission of the infection. This is crucial for hospitals to provide sufficient rooms, supplies, doctors, and medical personnel for successful treatment of all patients who need care. In this context, diagnostic tests play an essential role in control and surveillance of the novel COVID-19 outbreak. Moreover, timely and broad application of testing can lead to lower mortality rates, as, for instance, in Germany or South Korea (Morales-Narváez and Dincer, 2020).

Standard methods for emerging virus identification have been reviewed recently (Nguyen et al., 2020; Udugama et al., 2020; Younes et al., 2020) and are primarily based on real-time reverse transcription polymerase chain reaction (RT-PCR). Once the RNA (ribonucleic acid) sequence of SARS-CoV-2 was identified in January 2020 (Zhou et al., 2020), the WHO recommended the nucleic acid-based RT-PCR molecular diagnosis technique for SARS-CoV-2 detection from patients’ nasopharyngeal and/or oropharyngeal swab samples (WHO/COVID-19/laboratory/2020.5, 2020). The RT-PCR test is highly sensitive and detects even a tiny viral load in patients. However, the test is labor-intensive, requires skilled personnel, bulky and expensive equipment, is not suitable as a first-line screening tool or for on-site applications, and time-consuming (takes from 3 h up to 2–3 days including preparation of the viral RNA to give results) (Morales-Narváez and Dincer, 2020; Ozer et al., 2020; Ravina et al., 2020). For an effective outbreak containment, this time span is too long.

In order to overcome the limitations of RT-PCR-based systems and to facilitate massive diagnostic testing to counteract the increasing number of undetected cases, test manufacturers around the world have recently developed various portable/handheld, rapid, easy-to-use, point-of-care immunodiagnostic devices for on-site SARS-CoV2 detection in low-resource settings (e.g., in doctors’ practices or directly at home), each of which with its pros and cons (Morales-Narváez and Dincer, 2020; Nguyen et al., 2020; Ozer et al., 2020; Ravina et al., 2020; Udugama et al., 2020; Younes et al., 2020). These simple test kits are mostly based either on the detection of virus proteins in respiratory samples (e.g., sputum and throat swab), or of antibodies in human blood/serum, generated by the immune system in response to infection. However, based on current data, the WHO recommends the use of these new immunodiagnostic tests only in research settings and not yet for clinical decision-making, until evidence supporting their use for specific indications is available (WHO Scientific Brief, 2020). Therefore, there is an urgent need for new diagnostic tests and biosensors for virus detection, which are faster, more sensitive, accurate and reliable, easier, and more cost-efficient than existing ones (Bhalla et al., 2020). Such devices should also be capable of label-free, real-time detection/identification of viruses in clinical samples without or with minimal sample preparation steps, making on-site and in-field testing of a larger number of people possible within a shorter time period. Due to the small size, fast response time, label-free operation without need for expensive and time-consuming labeling steps, the possibility of real-time and multiplexed measurements, robustness and compatibility with advanced micro- and nanofabrication technology, biosensors based on semiconductor field-effect devices (BioFEDs) are one of the most fascinating platforms for an electrical detection of charged biomolecules and bioparticles by their intrinsic charge (Poghossian et al., 2013, 2015; Poghossian and Schöning, 2014; Yang and Zhang, 2014; Veigas et al., 2015; Syu et al., 2018). In this review, recent advances and key developments in the field of label-free detection of viruses (including plant viruses) with various types of BioFEDs are presented. Plant viruses are additionally introduced as promising bionanotools and building blocks of smart materials (e.g., Mao et al., 2009; Culver et al., 2015; Khudyakov and Pumpens, 2016; Koch et al., 2016; Wen and Steinmetz, 2016; Dragnea, 2017; Steele et al., 2017; Chu et al., 2018a; Lomonossoff, 2018; Lomonossoff and Wege, 2018; Wege and Lomonossoff, 2018; Chen et al., 2019; Eiben et al., 2019; Wege and Koch, 2020; Wen et al., 2020) that may bring about novel options for biosensor technology if applied as model particles, signal-amplifying colloids or, most importantly, multivalent adapter templates for the high surface-density presentation of detector components.

## Functioning Principle of BioFEDs

Although, at present, numerous BioFEDs based on an electrolyte-insulator-semiconductor (EIS) system have been developed using different sensor configurations, sensitive materials and fabrication technologies, the transducer principle of using an electric field to create regions of excess charge in a semiconductor is common to all of them. In this context, ion-sensitive field-effect transistors (ISFET) (Moser et al., 2016; Syu et al., 2018), extended-gate ISFETs (Pullano et al., 2018), capacitive EIS sensors (Poghossian et al., 2011; Bronder et al., 2015, 2019), light-addressable potentiometric sensors (Yoshinobu et al., 2001, 2017; Wu et al., 2016), silicon nanowire FETs (SiNW-FET) (Patolsky et al., 2006; Wang et al., 2016; Ambhorkar et al., 2018), graphene-based FETs (G-FET) (Choi et al., 2017; Syu et al., 2018), and carbon nanotube-based FETs (CNT-FET) (Choi et al., 2017; Alabsi et al., 2020) modified with biological recognition elements or receptors [e.g., enzymes, antibodies, antigens, peptides, DNA (deoxyribonucleic acid), and living cells] are typical examples of BioFEDs. During the last few years, label-free sensing of molecules by their intrinsic charge has become one of the most reported applications for BioFEDs (Poghossian and Schöning, 2014; Wu et al., 2015; de Moraes and Kubota, 2016; Kaisti, 2017; Bronder et al., 2018). Since FEDs are surface-charge-sensitive devices and because the vast majority of biomolecules are charged under physiological conditions, BioFEDs represent a universal platform for label-free electrostatic detection of a large variety of biomolecules and bioparticles including viruses. In the following, functioning of BioFEDs is briefly explained using the example of SiNW-FETs, which currently receive tremendous interest in biosensor design.

**FIGURE 1:**
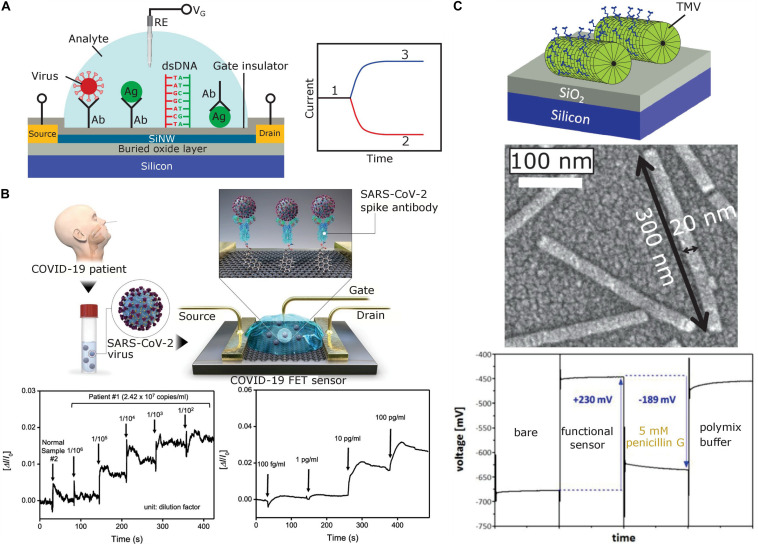
**(A)** Typical structure of a SiNW-FET prepared on a silicon-on-insulator wafer (left) and expected shift of original sensor signal (1) for a p-type SiNW-FET after binding of positively (2) or negatively charged (3) biomolecules (right). The SiNW-FET is composed of an active top thin Si layer covered with a gate insulator, source and drain electrodes, a thick buried SiO_2_ layer, and a bulk Si substrate. To selectively recognize target biomolecules or bioparticles, the gate insulator surface is functionalized with respective receptors (e.g., antibodies, antigens or ssDNA). **(B)** Schematic diagram of the COVID-19 FET sensor operation procedure (top), real-time response of COVID-19 FET toward SARS-CoV-2 cultured virus (bottom left), and SARS-CoV-2 antigen protein (bottom right). Adapted from Seo et al. (2020) with permission of the American Chemical Society. **(C)** Capacitive field-effect EIS sensor modified with TMV particles (top), scanning electron microscopy image of TMV particles on the sensor surface [middle, adapted from Poghossian et al. (2018) with permission from Elsevier], and constant-capacitance response of the EIS sensor [bottom, adapted from Koch et al. (2018a) with Creative Commons Attribution License]. Ab, antibody; Ag, antigen; RE, reference electrode; *V*_*G*_, gate voltage.

The typical structure of a SiNW-FET biosensor is illustrated in Figure 1A, where the channel region in a top Si nanowire between source and drain electrodes serves as the active sensing component. A gate voltage (*V*_*G*_) is applied via the third capacitively coupled electrode (reference electrode) to regulate the channel conductivity, working point and sensitive characteristics of the SiNW-FET. In order to selectively recognize target biomolecules or bioparticles in solution, the gate insulator surface of the SiNW-FET is functionalized with respective receptors [e.g., antibodies or single-stranded (ss) DNA probes]. The electric potential or charge changes at the SiNW-FET surface induced via the adsorption or binding of charged target biomolecules will alter the density of charge carriers in the channel and will, thus, modulate the conductivity of the channel and current between source and drain terminals. For a p-type SiNW-FET, by binding of positively charged biomolecules or bioparticles on the sensing gate surface, a depletion of charge carriers (in this case, holes) occurs in the nanowire channel. This will decrease the SiNW conductance and current in the nanowire channel for a fixed voltage between drain and source. Conversely, binding of negatively charged biomolecules induces an accumulation of holes, thus increasing the SiNW conductance and current. The opposite changes will be observed for n-type SiNW-FETs. For more detailed information concerning the operation principle and applications of BioFEDs, see reviews (Schöning and Poghossian, 2006; Poghossian and Schöning, 2014; Wang et al., 2016; Choi et al., 2017; Kaisti, 2017; Syu et al., 2018).

## Label-Free Detection of Viruses With BioFEDs

The strategies for label-free electrical detection/identification of viruses with BioFEDs can be subdivided into four categories: (1) direct identification of intact virus particles (virions) via the complete particle charge; (2) detection of viral antigens including non-virion proteins; (3) detection of viral nucleic acids (RNA or DNA); and (4) detection of antibodies produced by the immune system to identify and counteract or neutralize substances foreign to the body. The detection mechanism is always based on the direct measurement of changes in the electrical characteristics of BioFEDs caused from the binding events. The methods based on detection of intact virus particles, certain viral antigens and nucleic acids are more suitable for diagnosing new infected cases, while antibody detection techniques (serological tests) are better suited to determine whether an individual has previously been infected.

To date, different kinds of BioFEDs functionalized with various recognition elements have been applied successfully for the detection of numerous dangerous viruses. Some recent results reported in the literature are summarized in Table 1, which also includes the virus type, target, transducer, measurement range and lower detection limit. Selected key developments, including current results on detection of SARS-CoV-2 with BioFEDs, are discussed below.

**TABLE 1:** Selected examples of virus detection with different kinds of BioFEDs.

Virus, subtype	Target	Transducer	Measurement range	Lower detection limit	References
Influenza A, H1N1	Virus particle	Dual-channel FET	10^0.5^–10^8.5^ TCID_50_/mL	10^0.5^ TCID_50_/mL	Hideshima et al., 2019
		Nanonet FET	0.01–100 ng/mL	10 pg/mL	Park et al., 2019
		SiNW-FET	n.s.	∼3 × 10^4^ particles/mL	Shen et al., 2012
	Nucleic acid	SiNW-FET	2–102 pM	40 pM	Karnaushenko et al., 2015
Influenza A, H3N2	Virus particle	SiNW-FET	n.s.	∼3 × 10^4^ particles/mL	Shen et al., 2012
Influenza A, H5N2	Virus particle	SiNW-FET	10^4^–10^7^ particles/mL	10^4^ particles/mL	Chiang et al., 2012
Influenza A, H5N1	Virus particle	FET	10^0.5^–10^8.5^ TCID_50_/mL	10^0.5^ TCID_50_/mL	Hideshima et al., 2019
	Viral antigen	FET	10 pM–10 nM	5.9 pM	Kwon et al., 2020
	Nucleic acid	CNT-FET	1 pM–100 nM	1.25 pM	Thu et al., 2013
Influenza A	Virus particle	SiNW array	n.s.	5 × 10^4^ particles/mL	Patolsky et al., 2004
	Nucleic acid	SiNW-FET	1 fM–10 pM	n.s.	Lin et al., 2009
		SiNW-FET	n.s.	100 pM	Lin et al., 2012
		CNT-FET	1 pM–10 nM	1 pM	Tran et al., 2017
	Antibody	SiNW-FET	0.4–4 μg/mL	∼1 nM	Kim et al., 2014
		SiNW-FET	n.s.	20 μg/mL	Ahn et al., 2015
		FET	50 ng/mL–10 μg/mL	n.s.	Gu et al., 2009
Dengue	Nucleic acid	SiNW-FET	n.s.	2 fM	Nuzaihan et al., 2016
		SiNW-FET	n.s.	10 fM	Nuzaihan et al., 2018
		SiNW-FET	1–100 fM	∼10 fM	Zhang et al., 2010
	Viral antigen	FET	0.25–5 μg/mL	0.25 μg/mL	Vieira et al., 2014
HIV-1	Virus particle	G-FET	47.8 aM–4.78 fM	47.8 aM	Kim et al., 2019
HIV-2	Antibody	SiNW-FET	n.s.	4 μg/mL	Kim et al., 2014
Rotavirus	Virus particle	G-FET	10–10^4^ pfu/mL (fecal samples)	10^2^ pfu/mL	Liu et al., 2013
Ebola	Virus particle	G-FET	2.4 pg/mL–1.2 μg/mL	2.4 pg/mL	Jin et al., 2019
	Viral antigen	G-FET	1–444 ng/mL	1 ng/mL	Chen et al., 2017
SARS-CoV-2	Virus particle	G-FET	16–1.6 × 10^4^ pfu/mL (cultured virus) 242–24 × 10^4^ particles/mL (clinical samples)	16 pfu/mL 242 particles/mL	Seo et al., 2020
	Viral antigen	G-FET	1 fg/mL–10 pg/mL	1 fg/mL in buffer, 100 fg/mL in CTM	Seo et al., 2020
Hepatitis B	Nucleic acid	SiNW-FET	1 fM–1 pM	3.2 fM	Wu et al., 2014
Hepatitis C	Nucleic acid	CNT-FET	0.5 pM–5 nM	0.5 pM	Dastagir et al., 2007
Zika	Viral antigen	G-FET	n.s.	450 pM	Afsahi et al., 2018
Plum Pox (plant virus)	Virus particle	Organic FET	5 ng/mL–50 μg/mL	180 pg/mL (theoretical)	Berto et al., 2019

*TCID, 50% tissue culture infectious dose; n.s., not specified; pfu, plaque-forming unit; CTM, clinical transport medium for nasopharyngeal swabs.*

### Intact Virus Particle Detection

The viral load depends on the days after illness onset. Therefore, detection of intact virus particles can provide information to clinicians about the phase of the infection or therapy response. Viral particle concentration is often determined by way of plaque-based assays, by inoculating patient samples to cultivated cell lines and looking for cell death indicated by countable plaques in the confluent cell layer (Ozer et al., 2020). This method is, however, slow and is not applicable for point-of-care or on-site testing.

Since virus particles are generally charged in a wide pH range, BioFEDs are capable for the label-free electrostatic detection of adsorption or binding of charged intact virus particles onto their gate surface. To achieve specificity and to selectively capture the whole virus, the BioFED surface is usually functionalized with antibodies against specific surface proteins of the virus particle. To our best knowledge, the first direct and real-time detection of individual influenza A virus particles using antibody-functionalized SiNW biosensors was demonstrated in 2004 (Patolsky et al., 2004). The SiNWs were able to detect virus particles from a solution containing as low as 5 × 10^4^ particles/mL (Patolsky et al., 2004, 2006). In addition, a multiplexed simultaneous detection of different viruses (influenza A and adenovirus) using an array of individually addressable SiNWs was carried out. Thereafter, a lot of BioFED types have been designed and proven for the electrostatic detection of various intact virus particles, including several subtypes of influenza A [H1N1 (Hideshima et al., 2019; Park et al., 2019), H3N2 (Shen et al., 2012), H5N1 (Hideshima et al., 2019), H5N2 (Chiang et al., 2012)], human immunodeficiency virus (HIV) (Kim et al., 2019), rotavirus (Liu et al., 2013), Ebola (Jin et al., 2019), and SARS-CoV-2 (Seo et al., 2020). For instance, a SiNW biosensor for the rapid (within minutes) and low-cost diagnosis of seasonal flu that could detect H3N2 viruses in clinical, exhaled breath condensate samples down to ∼3 × 10^4^ particles/mL, was developed by Shen et al. (2012). An ultrasensitive detection of H5N2 avian influenza virus with a detection limit of 10^4^ viruses/mL (ca. 16 aM) has been demonstrated by a reusable SiNW-FET with reversible surface functionalization strategy (Chiang et al., 2012). More recently, a highly sensitive silicon-nanonet FET for the detection of H1N1 influenza A, which is one of the most virulent human pathogens among various types of influenza, was realized (Park et al., 2019). The nanonet FETs were able to detect H1N1 virus particles with a limit of detection down to 10 pg/mL (∼0.167 pM). Moreover, the detection and discrimination of human H1N1 and avian H5N1 influenza A viruses in nasal mucus samples by means of glycan-immobilized dual-channel FETs was discussed (Hideshima et al., 2019). To assess the feasibility of remote biosensing and to enable rapid information sharing, the biosensor system was connected to the smartphone via a Bluetooth connection.

In addition to SiNW-FET biosensors, G-FETs have been extensively studied for whole virus particle detection. For example, a G-FET based on micropatterned reduced graphene oxide was applied for real-time rotavirus detection (Liu et al., 2013). The lowest detection limit for rotavirus was determined as 10^2^ pfu/mL (plaque-forming units/mL), which is superior to conventional ELISA (enzyme-linked immunosorbent assay) tests. The biosensor was applied in fecal samples spiked with different concentrations (10–10^4^ pfu/mL) of rotavirus solution. A FET modified with reduced graphene oxide for the detection of inactivated Ebola virus particles from a spiked buffer solution with a detection limit of a 2.4 pg/mL was reported (Jin et al., 2019). Ebola virus is a highly pathogenic virus that invades most major organs and causes multisystem failure in humans with a case fatality rate of up to 90% (Jin et al., 2019). The biosensor with immobilized antibodies against the virus spike glycoprotein was successfully applied for the quantitation of inactivated Ebola viruses diluted in human serum, with a high specificity and a low detection limit of 12 pg/mL. An attomolar (47.8 aM) detection of HIV by coplanar-gate G-FETs prepared on flexible plastic substrates was demonstrated by Kim et al. (2019). Finally, quite recently, highly sensitive G-FETs have been engineered to determine the SARS-CoV-2 viral load in clinical nasopharyngeal swab samples from COVID-19 patients by way of graphene sheets coated with an antibody specific for the viral spike protein (Seo et al., 2020; see Figure 1B). The novel SARS-CoV-2 virus is highly infectious with a particle diameter of 60–140 nm (Scheller et al., 2020). The G-FET was successfully applied for label-free electrostatic and rapid detection of SARS-CoV-2 in culture medium and clinical samples (without sample pre-treatment) with a detection limit of 16 pfu/mL and 242 particles/mL, respectively (Seo et al., 2020).

### Detection of Viral Antigens

Biosensors for the detection of viral proteins represent immunologically sensitive FEDs (ImmunoFEDs), which detect affinity binding of viral antigens in a sample (e.g., from the respiratory tract of a person) to specific antibodies, antibody fragments or fusion proteins, affibodies or aptamers immobilized onto the gate surface, generating a detectable electrical signal (Poghossian and Schöning, 2014; de Moraes and Kubota, 2016). ImmunoFEDs for the label-free viral antigen detection were used to identify influenza A (Hideshima et al., 2013; Uhm et al., 2019; Kwon et al., 2020), Ebola (Chen et al., 2017; Generalov et al., 2019), dengue (Vieira et al., 2014), Zika (Afsahi et al., 2018), and SARS-CoV-2 (Seo et al., 2020) viruses. For example, a SiNW-FET functionalized with respective antibodies as receptor molecules was utilized for the detection of Ebola virus VP40 matrix proteins (Generalov et al., 2019). Extended-gate FET immunosensors were applied for the label-free detection of dengue virus non-structural proteins (Vieira et al., 2014) as well as of hemagglutinin glycoproteins of the highly pathogenic avian influenza virus H5N1 with a detection limit of 5.9 pM (Kwon et al., 2020). Moreover, attomolar detection of influenza A virus antigens with a glycan-modified FET was demonstrated (Hideshima et al., 2013). Real-time, quantitative detection of Zika viral antigens with a detection limit of 450 pM in buffer solution using commercially available graphene biosensor chips was reported (Afsahi et al., 2018). The potential of the biosensor for diagnostic applications was demonstrated by measuring the Zika antigen in diluted human serum samples. A G-FET modified with Au nanoparticles functionalized with anti-Ebola antibodies for real-time, highly sensitive and specific detection of the Ebola virus glycoprotein with a detection limit down to 1 ng/mL was developed as well (Chen et al., 2017). The applicability of this G-FET for point-of-care applications was evaluated in diluted buffer, human serum, and plasma spiked with Ebola glycoproteins. More recently, a G-FET biosensor was developed for the detection of SARS-CoV-2 spike proteins (Figure 1B; Seo et al., 2020). As receptor layer, specific antibodies against the SARS-CoV-2 spike glycoprotein were immobilized on graphene sheets [i.e., two-dimensional (2D) sheets of hexagonally arranged carbon atoms]. SARS-CoV-2 encodes four structural proteins (spike, envelope, membrane, and nucleocapsid), 16 non-structural proteins and nine accessory factors (Fehr and Perlman, 2015; Gordon et al., 2020). Among those, the spike proteins exposed on the virion surface are highly immunogenic and elicit specific antibodies best suited as reliable diagnostic markers for the immunodetection of a productive virus infection (Meyer et al., 2014; Mavrikou et al., 2020). The G-FET could detect the SARS-CoV-2 spike proteins with a detection limit of 1 fg/mL in phosphate-buffered saline and 100 fg/mL in clinical transport medium used for nasopharyngeal swabs. Moreover, the biosensor could distinguish the SARS-CoV-2 antigen protein from that of MERS-CoV (Seo et al., 2020). The authors claim that their biosensor can detect viral antigens in clinical samples without any preparation steps.

BioFEDs for the detection of viral antigens including virion and non-virion proteins could potentially be used for the rapid identification of infected patients, reducing or eliminating the need for expensive molecular confirmatory testing for viral nucleic acids. Antigen tests may thus be one way to scale up testing capacities to much greater levels. On the other hand, antigen tests are reliable only if the target viral proteins expressed by the virus are present in a sample in sufficient concentrations (i.e., when the respective gene products accumulate to detectable titers upon active virus replication). Swabs of patients (especially, for asymptomatic patients) infected with respiratory viruses often lack enough antigen material to be detectable. If, however, suitable antigens have been determined, their immunology-based detection is an excellent method allowing the identification of acute or early infection (WHO Scientific Brief, 2020).

### Detection of Virus Nucleic Acids

Most DNA BioFEDs are based on the detection of DNA-hybridization events and are constructed by immobilizing ssDNA capture probes onto the gate surface of the FED (Poghossian and Schöning, 2014; Bronder et al., 2015; Mu et al., 2015). During the DNA hybridization process, target DNA or RNA, respectively, within a sample is identified by a probe ssDNA that forms a double-stranded (ds) DNA or DNA/RNA helix with two reverse-complementary strands. Since nucleic acids are negatively charged in near-neutral aqueous solution, the additional charge associated with the hybridization-captured target molecule will effectively alter the gate surface charge, modulating the output signal of the BioFED.

Viral particles include either an RNA or a DNA genome of ss or ds nucleic acids. Single-stranded viral nucleic acids may exist in positive (+) sense, i.e., directly translatable, or negative (−) sense, i.e., complementary polarity. Therefore, a variety of BioFEDs (mostly based on SiNW-FETs or CNT-FETs) have been developed for detecting nucleic acid sequences of different viruses directly or after reverse transcription, including the genomic RNAs of influenza A virus [(−)ssRNA] (Lin et al., 2009, 2012; Kao et al., 2011; Thu et al., 2013; Karnaushenko et al., 2015; Tran et al., 2017), dengue virus [(+)ssRNA] (Zhang et al., 2010; Nuzaihan et al., 2016, 2018), or (+)ssRNA of hepatitis C virus (Dastagir et al., 2007), and the partially ds DNA of hepatitis B viruses (Wu et al., 2014; Lu et al., 2016). These BioFEDs are highly sensitive with detection limits often in the pM range, although a detection limit in the fM range was reported for ultrasensitive SiNW-FETs as well (Lin et al., 2009; Wu et al., 2014; Nuzaihan et al., 2016). However, most of the DNA probe-based BioFEDs have been tested in buffer solutions using short synthetic DNA sequences as model targets.

In spite of the ultrahigh sensitivity of SiNW-FET DNA biosensors reported in the literature, direct detection of unamplified nucleic acid in clinical specimens is still very difficult due to several reasons. To detect nucleic acids from real samples, the virus particles need to be disrupted (via heating or chemical treatment) in order to release the nucleic acid, which adds additional sample preparation procedures. Moreover, the amount of virus genomes present in clinical samples is in many cases far below the lower detection limit of reported DNA BioFEDs. Therefore, viral low-titer nucleic acids often demand for pre-amplification, e.g., by PCR using suitable primers. To enable a reliable detection of virus RNA [including the (+)RNA of SARS-CoV-2] from clinical samples without additional amplification, the detection limit of nucleic acid biosensors should be below ∼100 aM (Ozer et al., 2020). Otherwise, RT-PCR techniques may be used for reverse transcription of virus RNA extracted from patient samples into complementary cDNA, and amplification of target sequences from the resulting cDNA template. One example of such concept is a silicon-based microfluidic system combining a chip-based PCR module for amplification of nucleic acid targets, and a multiplexed SiNW sensing module developed for detection and differentiation of influenza A strains (swine-originated H1N1 and seasonal Flu A) according to sequence variations in the viral (−)RNA as identified through the corresponding cDNAs (Kao et al., 2011). Highly appealing due to their convincing sensitivity, speed and easy use even without pricey laboratory equipment are isothermal amplification techniques such as reverse-transcription-loop-mediated isothermal amplification (RT-LAMP; Russo et al., 2020) and recombinase polymerase amplification (RPA; Esbin et al., 2020).

Taken together and due to the low sample consumption, high sensitivity and specificity, such chip-based PCR or isothermal amplification modules in combination with FED sensor systems could be attractive alternatives for point-of-care applications.

### Detection of Host Antibodies

Antibody tests are typically used to detect the presence of virus-specific antibodies (immunoglobulins) in the blood of virus hosts, when the immune system is responding to a particular infection (Younes et al., 2020). Immunoglobulin M (IgM) antibodies are usually produced during the onset of the infectious disease (between 4 and 10 days after virus uptake), whereas immunoglobulin G (IgG) responses occur later (around 2 weeks post inoculation) (Morales-Narváez and Dincer, 2020). Therefore, antibody detection tests can be useful to understand how many people have been exposed to a virus and underwent a symptomatic or asymptomatic infection (which is of primary importance for a better understanding of the SARS-CoV-2 epidemiology), as well as to support the development of vaccines.

Immuno-FEDs for the detection of specific host antibodies against viruses are prepared by an immobilization of viral capture antigens serving as receptors on the gate surface. Such biosensors detect charge changes, induced by affinity binding of host target antibodies to viral antigens. Immuno-FEDs for the detection of antibodies against viruses have been rarely studied (mostly as proof-of-concept experiments). For example, a nanogap FET (Gu et al., 2009), an underlap channel-embedded FET (Lee et al., 2010), and a SiNW-FET (Ahn et al., 2015) were realized to detect specific antibodies directed against avian influenza viruses. Moreover, the multiplexed detection of antibodies against avian influenza and human immunodeficiency viruses (HIV) by means of an underlap-embedded SiNW-FET is demonstrated (Kim et al., 2014). In another approach, a SiNW biosensor integrated with a microfluidic channel was applied for the detection of antibodies against Aleutian disease virus in serum samples from infected minks (Svendsen et al., 2011). Finally, an extended-gate FET was developed and tested for the detection of antibodies against bovine herpes virus-1 in both commercially available antiserum and real serum samples from cattle (Tarasov et al., 2016).

Summarizing this section, it should be noted that the stability of the reference electrode and the level of leakage current are crucial factors for a correct functioning of BioFEDs; they will essentially impact accuracy, reproducibility and reliability of measurements. In spite of this fact, in many papers discussed in the literature, information on type or stability of (quasi-) reference electrodes used, as well as on the leakage current level is missing.

## Detection of Plant Viruses as Pathogens and Potential Model Particles

Plant viruses are among the major contributors to economic losses in agriculture [more than 50 billion €/year worldwide (Pallás et al., 2018)]. Therefore, there is great interest in sensitive, rapid and easy-to-use portable devices for an early detection of viruses in infected plants by in-field or on-site application (Khater et al., 2017; Cassedy et al., 2020).

Notwithstanding, we have found only two cases of electrostatic detection of plant virus particles with FEDs. The usability of capacitive field-effect EIS sensors for label-free electrical detection of plant virus particles was initially demonstrated by Koch et al. (2018a) and Poghossian et al. (2018) for tobacco mosaic virus (TMV). Here, EIS structures with adsorbed TMV particles were used for designing a penicillin biosensor, where the TMV particles served as nanocarriers for enzymes installed at high surface densities on the viral coat protein (CP) subunits. TMV has a nanotube-like structure with a single RNA molecule and 2,130 CPs helically assembled into full-length particles of 300 nm, outer diameter of 18 nm and a longitudinal internal channel of 4 nm diameter (Klug, 1999; Scholthof et al., 1999; Culver, 2002; Lomonossoff and Wege, 2018; Wege and Koch, 2020). TMV is harmless for mammals (Nikitin et al., 2016) and lacks a membrane envelope; it and related tobamoviruses infect numerous plant species in several families through mechanical transmission fast and efficiently (Zaitlin, 2000; Adams et al., 2017). Tobacco (family *Solanaceae*) leaves systemically infected with wildtype TMV develop characteristic mosaic-like patterns, but symptoms in other plants and with TMV mutants may be less distinctive (Culver, 2002). Frequent outbreaks of TMV and related viruses in cultivated plants, namely in greenhouse crops such as tomato, pepper, cucurbits, and ornamentals thus demand for rapid identification to avoid substantial economic losses (Scholthof et al., 2011; Moriones and Verdin, 2020). This seems possible by way of FEDs: A single loading of TMV particles onto a Ta_2_O_5_-gate EIS sensor surface resulted in a large signal change of 230 mV (see Figure 1C; Koch et al., 2018a), which is associated with the negative charge of the TMV particles. The model study used biotinylated TMV, but should be valid also for native TMV particles exhibiting a similar charge. Recently, an electrolyte-gated organic FET biosensor for the quantification of plum pox virus (PPV) in plant extracts was realized (Berto et al., 2019). PPV is highly infectious, causes the devastating Sharka disease and thereby affects stone fruit trees in most parts of the world (Hajizadeh et al., 2019). Early PPV recognition is crucial to eliminate infected trees from orchards before the virus has been spread by its insect vectors (aphids) further. Anti-PPV polyclonal antibodies were immobilized by Berto et al. (2019) on the separated Au gate electrode. The biosensor shows great promise for in-field applications as it was able to detect specific binding of PPV particles to anti-PPV antibodies in plant extracts with a sub ng/mL detection limit.

These two incidences of FED-based plant virus detection do not only point to agronomically relevant perspectives for monitoring such viruses by label-free biosensors, they also demonstrate a huge potential of plant-harvested viruses as harmless model and calibration particles for the electrical detection of animal and human viral diseases. For a plenitude of differently shaped and charged plant-borne viruses, purification and storage protocols have been optimized during the last decades (Dijkstra and de Jager, 1998; Wege and Lomonossoff, 2018). Furthermore, several robust plant viruses are employed already commercially for the production of recombinant virus-like particles (VLPs) displaying domains of non-plant viral proteins on their outer CP surfaces, including SARS-CoV-2 epitopes (Capell et al., 2020; Rosales-Mendoza, 2020). Such preparations could be of high value for the development of FED formats suitable to pre-select or identify COVID-19-infected samples from patients, by help of plant-derived mimics of SARS-CoV-2 that serve as model particles to determine appropriate FED setups and detection conditions.

## Plant Virus-Based Building Blocks Enhancing Biosensor Performance

Since more than two decades, several plant viruses attract increasing attention also from a different point of view: Their precise and robust nanostructures with repetitively organized, multivalent protein surfaces lend these viruses and derivatives thereof to uses in medical and technical environments, as carrier particles for the delivery and/or display of functional units enclosed and/or exposed at high densities (Bittner et al., 2013; Lin and Ratna, 2014; Culver et al., 2015; Khudyakov and Pumpens, 2016; Koch et al., 2016; Wen and Steinmetz, 2016; Dragnea, 2017; Steele et al., 2017; Lomonossoff and Wege, 2018; Wege and Lomonossoff, 2018; Balke and Zeltins, 2019; Chen et al., 2019; Eiben et al., 2019; Roeder et al., 2019; Chung et al., 2020; Wege and Koch, 2020; Wen et al., 2020). The respective plant viruses and VLPs are richly and sustainably available by farming (Marsian and Lomonossoff, 2016; Gowtham and Sathishkumar, 2019; Rybicki, 2020), and despite a remarkable durability biodegradable after use. Certain plant viral CPs are amenable to modifications facilitating the selective coupling of functional molecules, and to *in vivo* or *in vitro* assembly into VLPs even in the absence of viral nucleic acids (Wege and Koch, 2020). This allows the fabrication of artificial, bioinstructive carrier particles of adapted shapes and surface chemistries. These benefits of plant virus-based immobilization templates might offer novel options for improving SARS-CoV-2 biosensors, in analogy to promising results with previously developed virus nanoparticle-assisted detection systems.

### Applicability of Plant Viral Nanoscaffolds

On account of the properties sketched above, many biomedical uses of plant viral nanoparticles are emerging and have been reviewed in detail (Franzen and Lommel, 2009; Czapar and Steinmetz, 2017; Steele et al., 2017; Aljabali et al., 2018; Eiben et al., 2019; Hema et al., 2019; Benjamin et al., 2020; Chung et al., 2020; Silva et al., 2020). They include, among others, the directed delivery of imaging agents and therapeutics to target sites, e.g., via the blood stream to tumors or atherosclerotic lesions. Cell culture and tissue engineering were shown to profit from cell adhesion and differentiation-mediating peptides presented on plant viral scaffolds in 2D and three-dimensional (3D) layouts. The largest and most advanced area of medical uses are plant VLP-based self-adjuvanting vaccines (Chackerian, 2007; Crisci et al., 2012; Matić and Noris, 2015; Hefferon, 2018; Balke and Zeltins, 2020; Rybicki, 2020; Santoni et al., 2020) with candidates against COVID-19 in the developmental pipelines of at least two companies (Rosales-Mendoza, 2020), as specified also in this research topic. Similarly, plant viral particles are being evaluated on various technological platforms that gain enhanced or even novel functionality through an integration of multivalent, selectively addressable bionanostructures (Fan et al., 2013; Culver et al., 2015; Koch et al., 2016; Dragnea, 2017; Narayanan and Han, 2017; Chu et al., 2018a; Chen et al., 2019; Wege and Koch, 2020). Uses as templates for inorganic and synthetic compounds have led to biohybrid materials of convincing properties (Douglas and Young, 1998; Bittner et al., 2013; Vilona et al., 2015; Tiu et al., 2016; Wen and Steinmetz, 2016; Lee et al., 2017; Zhang et al., 2018; Eiben et al., 2019), such as high-capacity battery electrodes or spatially ordered dye ensembles for light-harvesting. If employed as immobilization scaffolds for biomolecules, from peptides and antibodies up to enzymes, plant VLPs exhibit special advantages (Sapsford et al., 2006; Werner et al., 2006; Comellas-Aragones et al., 2007; Minten et al., 2011; Aljabali et al., 2012; Pille et al., 2013; Uhde-Holzem et al., 2016; Roeder et al., 2017; Dickmeis et al., 2018; Koch et al., 2018b; Tian et al., 2018; Yuste-Calvo et al., 2019a; Aves et al., 2020; Park et al., 2020). This has laid the foundation for novel plant virus-supported biocatalytic nanomaterials (Carette et al., 2007; Cardinale et al., 2012; Koch et al., 2015; Besong-Ndika et al., 2016; Cuenca et al., 2016; Brasch et al., 2017; Schwarz et al., 2017; Aumiller et al., 2018; Chakraborti et al., 2019), and for biodetection formats that may serve as blueprints for novel SARS-CoV-2 sensor layouts, as outlined in the following.

### Plant Virus-Enhanced Biosensors: State-of-the Arts and Perspectives

Plant viral soft-matter nanoparticles with hundreds up to thousands CP subunits offer one-of-a-kind opportunities for enhancing the performance of miniaturized biosensors including BioFEDs for a fast, reliable, durable and economically reasonable on-site detection of many targets (Mao et al., 2009; Koch et al., 2016; Lomonossoff and Wege, 2018; Eiben et al., 2019; Benjamin et al., 2020; Wege and Koch, 2020). Natural and engineered viral CPs allow selective coupling of biorecognition elements by direct or linker-mediated chemical conjugation and/or affinity docking, and in some cases genetic or (auto-)catalytic fusion (see references above). Thereby, capture units such as antibodies, trappable peptides or target-specific single-type or cooperating enzymes can be installed at down to nanometer distances in monolayers or staggered arrangements on the viral backbones. In turn, plant viral adapter scaffolds may be immobilized efficiently on different types of sensor surfaces. Various deposition techniques for viruses and VLPs to bare and pre-treated technical surfaces have been optimized, including adsorption, spin- and convective coating, intermediate self-organization at liquid/liquid interfaces (Wege and Lomonossoff, 2018; Zhang et al., 2018), electrokinetics such as electrophoresis and dielectrophoresis (Lapizco-Encinas and Rito-Palomares, 2007; Bittner et al., 2013), electrowetting (Chu et al., 2018b) or microfluidics (Zang et al., 2017). They can be applied to viral nanoparticles before or after their loading with target recognition elements to yield receptor layers of high surface densities, which may increase sensor sensitivity substantially in comparison to conventional layouts. Such “ultradense” presentation of efficiently immobilized capture units by way of plant VLP adapter templates has been demonstrated in several biosensor layouts for distinct systems with both indirect and label-free read-out (e.g., Szuchmacher Blum et al., 2011; Zang et al., 2014, 2017; Fan et al., 2015; Koch et al., 2015, 2018a; Tinazzi et al., 2015; Bäcker et al., 2017; González-Gamboa et al., 2017; Poghossian et al., 2018; Yuste-Calvo et al., 2019b), as also detailed in the respective sections of recent reviews (Koch et al., 2016; Eiben et al., 2019; Benjamin et al., 2020).

For COVID-19 diagnostics by point-of-care devices, highest detection sensitivities through SARS-CoV-2 enrichment on densely antibody- or aptamer-equipped sensors will be crucial to minimize false-negative results in swab samples from early or late infection stages, and in diluted gargle lavages (“mouthwashes”) (Malecki et al., 2020) increasingly utilized for convenient high-throughput testing. Plant virus interlayers on sensor chips may be of high practical value in this context. Furthermore, robust plant VLPs can also serve as additional signal-amplifying colloids if applied post target trapping on a sensor surface. In this case, bifunctional VLPs displaying both capture and signal-generating elements are suitable for indirect sensor layouts, like ELISA or fluorescent microchip arrays (Soto et al., 2006, 2008, 2009), whereas direct, label-free sensors including BioFEDs can make use of VLPs equipped with target capture units only.

Last but not least, different sensor systems with biorecognition elements exposed on plant viral carriers were shown to harbor unexpectedly enhanced reusability and long-term stability over weeks up to months, in comparison to their plant virus-free counterparts (Koch et al., 2015, 2018a). Enzyme-based, TMV-assisted capacitive field-effect EIS sensors for antibiotics retained full sensitivity over at least one year of repeated uses (Poghossian et al., 2018). They were also compatible with “real-world” samples, i.e., diluted milk. Amperometric glucose sensors with TMV nanocarriers for glucose oxidase did not only exhibit higher sensitivity than the sensors devoid of TMV, they also had faster response time and extended linear detection range (Bäcker et al., 2017). However, an electrical detection of coronaviruses in combination with plant VLP-immobilized SARS-CoV-2-specific antibodies, synthetic recognition elements or virus nucleic acid-directed probes, respectively, yet remains to be evaluated, preferably in a BioFED layout. Against the background of the above and several other examples, advantageous sensor properties conveyed by the plant viral carrier templates are likely, with respect to shelf-life, sensor robustness, reusability and overall performance. To evaluate and establish options for routine applications in commercially available devices, it seems crucial to define globally harmonized regulatory prerequisites and standardized rules of good manufacturing practice (GMP).

## Conclusion

Viral diseases are one of the major threats to health and life of the world population. Early diagnosis and treatment are essential in order to slow down the virus spread and to contain the disease outbreak. The SARS-CoV-2 pandemic has dramatically highlighted the critical role of diagnostic technologies in the control of infectious diseases. Hence, development of new rapid, highly sensitive, accurate and reliable, easy-to-use and cost-efficient, portable point-of-care diagnostic tests, and devices for virus detection in low-resource settings has tremendous importance for medical healthcare. In this review, recent advances and key developments in the field of a label-free detection of various dangerous viruses by means of different types of BioFEDs, which represent one of the most promising transducer platforms for miniaturized biosensors, are presented.

The study of the current state of BioFEDs for virus detection reveals that BioFEDs, especially SiNW-FETs and G-FETs, enable an ultrasensitive label-free electrical detection of intact virus particles (including plant viruses), viral antigens and nucleic acids as well as antibodies against viruses by their intrinsic charge. For some BioFEDs, a detection limit down to the fM concentration range has been reported. In addition, multiplexed detection and discrimination of viruses was demonstrated. Other advantages of BioFEDs are small sizes, fast response time and the possibility of real-time detection on the one hand, and the possibility of integration with on-chip microfluidics and compatibility with complementary metal-oxide-semiconductor (CMOS) technology allowing the fabrication of large volumes of reproducible devices with lower costs on the other hand. Moreover, the optional detection of more than one virus-related parameter (intact virus particle, viral antigen, viral nucleic acids, and antibodies generated against virus) with an array of BioFEDs on the same chip could offer more accurate and reliable disease diagnosis.

Despite remarkable progress in BioFEDs for label-free virus detection, it should be noted that BioFEDs are often studied under rather ideal experimental conditions. There are still some limitations (e.g., screening the charge of biomolecules or virus particles by counter ions in the solution, or possible non-specific binding of further biomolecules present in samples on the sensor surface) for BioFED applications with real biological samples (whole blood, plasma, serum, urine, saliva, nasopharyngeal swabs, or gargle lavage) that must be overcome, before their transfer from scientific laboratories to real life will appear. Biological samples contain a large number of charged chemical species, which are able to non-specifically adsorb on the gate surface of the FEDs, generating a false-positive signal or masking the useful signal from the target of interest. This could substantially hamper the sensitivity, specificity and reliability of FEDs. Therefore, recently, several different strategies have been proposed to reduce the influence of the counter-ion screening effects (e.g., the use of desalted/filtered samples or short receptors) or non-specific adsorption (the use of blocking agents, pre-filtering/purifying the biological liquids or on-chip separation and pre-concentration). Thus, the sensitivity and detection limit of FEDs can be distinctly enhanced. Another task is the development of a stable and reliable, miniaturized reference electrode integrated onto the FED chip. In addition, the reproducibility of surface modification and receptor immobilization procedures, specificity, stability, time-to-result and reliability of FEDs represent further key parameters, which need to be improved for “real life” measurements. Generally, a real-time label-free electrostatic detection of charged molecules and biological particles in untreated biological samples still remains challenging. The success in implementation and widespread application of FEDs for virus detection will depend on how advanced they are compared with the current gold standards in terms of simplicity, rapidity, sensitivity, specificity and reliability.

In this context, plant viruses and VLPs applied as nanocarriers for target recognition elements offer exciting perspectives for enhancing the performance of miniaturized on-site biosensors with regard to stability, functionality in complex sample mixtures, sensitivity, and further detection parameters. Their endurance may obviate the need for frequent re-calibration of handheld devices, which would be of particular importance for COVID-19 and other viral diseases’ early detection in developing countries and remote regions. Sustainably produced by farming, and by increasing the operating life of sensor chips, they also avoid wastage of energy and resources and may thus give new impetus to the development of powerful “biologized” smart mini-tools. Plant virus-assisted BioFED sensors could therefore be among the high-priority developments in the multi-toolbox currently worked out to disarm SARS-CoV-2. Time seems high to harmonize international regulations for the use of plant viral building blocks in technical devices (see also Eiben et al., 2019), including both natural types and genetically engineered variants that enable simplified technical applications as carrier/adapter platforms due to, e.g., increased numbers of easily addressable docking sites for analyte-specific receptors displayed on the plant viral particles.

In summary, we believe that BioFEDs, plant viruses and combinations thereof can play significant roles in point-of-care and on-site testing for an early diagnosis and treatment of infectious diseases in the future.

## Author Contributions

All authors listed have made a substantial, direct, and intellectual contribution to the work and approved it for publication.

## Conflict of Interest

The authors declare that the research was conducted in the absence of any commercial or financial relationships that could be construed as a potential conflict of interest.
